# Identification of SH3 Domain Proteins Interacting with the Cytoplasmic Tail of the A Disintegrin and Metalloprotease 10 (ADAM10)

**DOI:** 10.1371/journal.pone.0102899

**Published:** 2014-07-18

**Authors:** Henriette Ebsen, Marcus Lettau, Dieter Kabelitz, Ottmar Janssen

**Affiliations:** University of Kiel, Molecular Immunology, Institute for Immunology, University Hospital Schleswig-Holstein Campus Kiel, Kiel, Germany; Hungarian Academy of Sciences, Hungary

## Abstract

The a disintegrin and metalloproteases (ADAMs) play a pivotal role in the control of development, adhesion, migration, inflammation and cancer. Although numerous substrates of ADAM10 have been identified, the regulation of its surface expression and proteolytic activity is still poorly defined. One current hypothesis is that both processes are in part modulated by protein-protein interactions mediated by the intracellular portion of the protease. For related proteases, especially proline-rich regions serving as docking sites for Src homology domain 3 (SH3) domain-containing proteins proved to be important for mediating regulatory interactions. In order to identify ADAM10-binding SH3 domain proteins, we screened the All SH3 Domain Phager library comprising 305 human SH3 domains using a GST fusion protein with the intracellular region of human ADAM10 as a bait for selection. Of a total of 291 analyzed phage clones, we found 38 SH3 domains that were precipitated with the ADAM10-derived fusion protein but not with GST. We verified the binding to the cytosolic portion of ADAM10 for several candidates by co-immunoprecipitation and/or pull down analyses. Intriguingly, several of the identified proteins have been implicated in regulating surface appearance and/or proteolytic activity of related ADAMs. Thus, it seems likely that they also play a role in ADAM10 biology.

## Introduction

The a disintegrin and metalloprotease (ADAM) proteins form a subgroup of the metzincin superfamily that also comprises matrix metalloproteinases (MMPs) and the ADAM proteases with thrombospondin motifs (ADAMTSs). ADAMs are glycosylated type I transmembrane proteins that are specialized for juxtamembrane cleavage of spatially associated membrane proteins [1], [2]. This ectodomain shedding results in the release of bioactive extracellular protein fragments. For example, TNF-α is liberated by the TNF-α converting enzyme (TACE, ADAM17) [3] and the Fas ligand (FasL, CD95L) is cleaved by ADAM10 [4]–[6].

**Figure 1:**
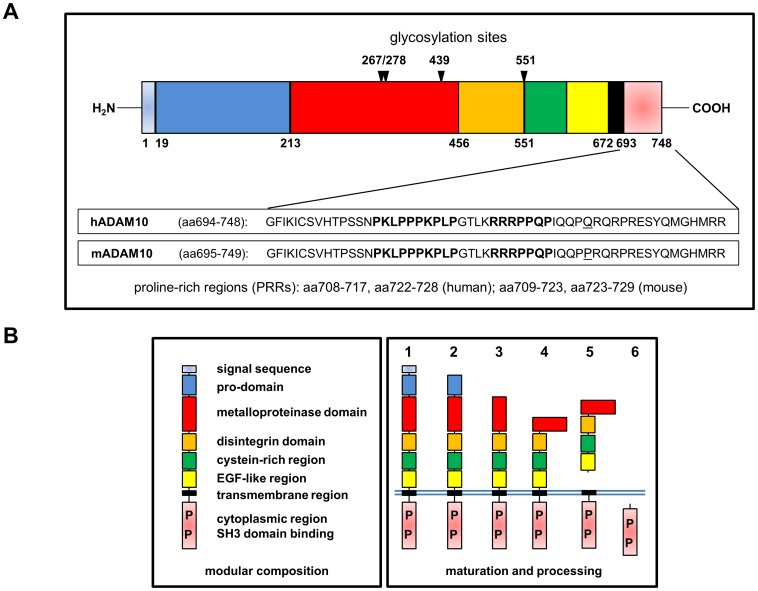
Schematic representation of the domain structure of ADAM10. (**A**) The cytoplasmic tail at the C-terminus harbors two proline-rich regions (PRR) that might enable binding to SH3 or WW domain-containing proteins. The intracellular parts of human and murine ADAM10 are highly conserved and differ in only one amino acid. (B) Modular composition, maturation and processing of ADAM10. The N-terminal signal sequence of the protease is needed for intracellular maturation (1→2). To generate an enzymatically active protease, the pro-domain has to be removed by a protein convertase such as furin (2→3). The catalytic metalloproteinase domain is the largest domain of ADAM10 and might be activated by different signals including substrate-induced conformational changes at the plasma membrane (3→4). The membrane-proximal region is important for adhesion and substrate recognition and contains a disintegrin, a cysteine-rich and an EGF-like domain. ADAM10 itself is subjected to proteolysis by ADAM9 or ADAM15 and the γ–secretase complex (4→5). The fate and functions of the soluble ectodomain or the resulting C-terminal fragments are still unclear. Notably, intracellular regions at all stages might interact with individual SH3 domain-containing interaction partners.

Structurally, ADAMs contain several distinct subdomains including a signal peptide, a pro-domain that is cleaved off during maturation, a metalloprotease domain, a disintegrin domain, a cysteine-rich region, an EGF (epidermal growth factor)-like or membrane-proximal domain, a transmembrane domain and an intracellular region (Fig. 1). Since only 13 of the 21 or 22 presumed functional human ADAMs possess proteolytic activity [1], [2], it is likely that other domains also contribute to the overall biological functions of ADAM proteins. For example, the disintegrin domains guide interactions with integrins and the cysteine-rich domains support cell adhesion by binding to syndecans or fibronectin or clustering with other ADAMs [7]. The C-terminal cytoplasmic tails vary in length between different members of the family and have been implicated in the regulation of ADAM maturation, activity and localization [8]. Different phosphorylation sites seem to be relevant for signal transduction in the context of ADAM mobilization or activity [1]. For some ADAMs, serine/threonine and/or tyrosine phosphorylation was reported [9], [10], which might lead to the generation of inducible binding sites and/or protein complex formation, for example to facilitate Src homology 2 (SH2) domain protein binding upon tyrosine phosphorylation.

In addition, most ADAM proteases contain one or more proline-rich stretches, that potentially enable interactions with Src homology 3 (SH3) domain-containing signaling molecules. As depicted in Fig. 1, except for one proline-to-glutamine exchange, the intracellular regions of ADAM10 are identical in mice and humans, suggesting an important and highly conserved regulatory function. In human ADAM10, the two prominent proline-rich regions (PRRs) comprise amino acids 708–717 (PKLPPPKPLP) and 722–728 (RRRPPQP), respectively. As mentioned, different ADAM family members vary in the number of intracellular PRRs. Whereas some ADAM proteins do not contain any classical SH3 domain binding site (e.g. ADAM28), other members contain only one or two (ADAM10, ADAM17) and some ADAMs (ADAM12, ADAM15) contain multiple SH3 binding motifs. This in turn suggests that specific PRR-mediated protein-protein interactions might exert characteristic functions to modulate transport, localization and/or regulation of activity of individual ADAM family members.

The first screening for ADAM-interacting SH3 domain proteins was performed by Cousin and colleagues who described the interaction of the protein kinase C and casein kinase substrate in neurons 2 (PACSIN2) and ADAM13 in *Xenopus* embryos/cells. Functionally, overexpression of X-PACSIN2 could rescue developmental alterations induced by overexpression of ADAM13 [11]. Given that individual SH3 domains of different proteins bind to several similar PRRs, two or even more putative SH3 domain binding sites indicate that individual ADAM proteases might associate with a whole array of SH3 domain-containing proteins. As an example, ADAM12 was reported to bind Src kinases (c-Src and Yes), the growth factor receptor-bound protein 2 (Grb2) [12], [13], the p85α subunit of phosphatidylinositol 3 kinase (PI3K) [14], the protein kinase C and casein kinase substrate in neurons 3 (PACSIN3) [15], α-Actinin-1 and -2 [16], [17], the adaptor protein TKS5 (Five SH3 domain-containing protein  =  FISH) [18] and the SH3 domain-containing protein 19 (SH3D19, ADAM-binding protein Eve-1, EEN-binding protein, EBP) [19]. Several of these interactions have been further investigated in different cellular systems. It turned out that PACSIN3 up-regulates ectodomain shedding of the ADAM12 substrate heparin-binding epidermal growth factor-like growth factor (HBEGF) [15], similar to what was described shortly thereafter for the multidomain adaptor protein Eve-1 by Tanaka and colleagues [19]. ADAM12-L, the long transmembrane form of ADAM12, transiently interacts with c-Src. When ADAM12-L gets phosphorylated by c-Src, it redistributes from perinuclear regions to actin-associated regions at the cell periphery. Interestingly, in response to external signals such as integrin engagement, or by overexpression, ADAM12-L in turn enhances Src kinase activity, most prominently in focal adhesions [20]. The interaction of ADAM12 with Grb2 apparently regulates internalization and recycling of the protease in a clathrin-dependent manner, whereas it does not affect protease activity [21]. For ADAM9 and ADAM15, Endophilin-A1 (EEN-B1, SH3GL1) and sorting nexin 9 (SNX9, SH3PX1) have been identified as putative interactors by yeast two-hybrid screens and their association was confirmed by pull down assays and co-immunoprecipitation [22]. Both Endophilin-A1 and SNX9 are implicated in endocytosis and intracellular protein trafficking processes [23]–[25]. ADAM15 also interacts with Grb2 and the Src family tyrosine kinases Src, Lck and Hck. Upon phosphorylation, ADAM15 shows an increased binding to Lck, Hck and Grb2 [10]. Moreover, an interaction of the SH3 domain-containing protein synapse-associated protein 97 (SAP97) with the cytosolic portion of ADAM10 was reported to modulate the localization and thereby the activity of ADAM10 [26]. An interaction of SAP97 was also shown before for ADAM17 [27]. These few examples illustrate the importance, the complexity and maybe the dynamics of SH3 domain-associated signaling networks in the context of ADAM proteases.

Given that ADAM10 and its closest relative ADAM17 are meanwhile regarded as key regulators of cell adhesion, migration, inflammation and cancer, they are also in focus as putative therapeutic targets [28], [29]. Thus, it is important to know how these proteases are regulated on a molecular level. An insight into their intracellular interactome might provide hints and ideas which signaling proteins modulate ADAM protease expression and activity. Based on analogy to other ADAM proteases, SH3 domain-mediated interactions with respective proline motifs within the intracellular region attracted our attention. We therefore used a library of human SH3 domain expressing phages contained in the All SH3 Domain Phager to identify proteins that potentially bind to the intracellular part of human ADAM10 expressed as a glutathione S-transferase (GST) fusion protein (GST-hADAM10(697–748)). From 291 sequenced clones, a surprising high number of 38 candidate proteins was identified whose SH3 domains precipitated with GST-hADAM10(697–748) but not with GST alone. Interestingly, many of the identified proteins (i.e. endophilins, non-receptor protein tyrosine kinases (PTK), Grb-2-related adaptor proteins, sorting nexins and PACSINs) fit into the categories of proteins described as interaction partners for other ADAM proteases such as ADAM9, 12 and 15. Of note, more or less all identified SH3 domain proteins have in common that they are somehow involved in membrane-to-cytoskeleton signaling, mostly affecting protein trafficking or localization. We therefore assume that several ADAM proteases are indeed regulated in a comparable manner by similar subsets of SH3 domain-containing proteins.

## Material and Methods

### Production of recombinant GST fusion proteins

Standard RT-PCR-based cloning strategies were used for the expression of the intracellular part of ADAM10 or ADAM17 as glutathione S-transferase (GST) fusion proteins. Here, cDNA from activated human T cell blasts served as a template. Primers for ADAM10 (forward 5′-TGGGATCCGGCAAGATATGCAGTGTTC-3′ and reverse 5′-TGGAATTCTTAG CGTCTCATGTGTCCC-3′) and ADAM17 (forward 5′-CAGGATCCGGTGGTGTG GATAAGAAATTG-3′ and reverse 5′-GGAATTCTTAGCACTCTGTTTCTTTGCTG-3′) were designed according to the published sequence (GenBank, accession number AAC51766 and AAI36784) with adding flanking BamH1 and EcoR1 restriction sites for unidirectional insertion of purified fragments encoding hADAM10(697–748) and hADAM17(694–824) into pGEX-2T (GE Healthcare, Freiburg, Germany). In frame insertion was verified by sequencing before transforming competent *E. coli* (DH5α). Individual clones were selected by small scale protein expression and visualization by coomassie staining after SDS-PAGE. Selected clones were then used for medium scale purification from culture supernatants. To this end, LB medium supplemented with ampicillin was inoculated with an overnight culture of GST-hADAM10(697–748)-/GST-ADAM17(694–824)-transformed or GST-transformed bacteria. After 1.5 hours, protein expression was induced by adding 0.1–0.5 mM isopropyl-β-D-1-thiogalactopyranoside (IPTG) for 4 hours. Bacteria were pelleted, resuspended in cold PBS, sonicated and further lysed by adding 1% (v/v) Triton X-100 in PBS. After centrifugation, the supernatant was incubated with glutathione sepharose 4B beads (GE Healthcare, Freiburg, Germany) at 4°C. The bound fusion protein was eluted with reduced glutathione and extensively dialyzed in PBS and concentrated to 1 mg/ml by centrifugation on Amicon filter units (Millipore, Billerica, USA). All other GST-tagged fusion proteins used for pull down experiments were produced and purified following the standard protocol as described elsewhere [30]–[32].

### Bacteria and phages

For the identification of ADAM10-interacting SH3 proteins, a commercially available M13 phage display library was used (All SH3 Domain Phager Geneart AG, Regensburg, Germany). For the screening, *E. coli* TG1 bacteria (Stratagene, La Jolla, USA) were inoculated on M9 plates and expanded in M9-glu medium with high glucose content overnight. Afterwards, an equivalent of OD (λ = 600 nm)  = 0.05 was diluted in 50 ml sterile LB medium. Following cultivation at 37°C, transduction competence of the bacteria was checked by infecting the bacteria with M13K08 helper phages (New England Biolabs, Ipswich, USA). The acquired kanamycin resistance revealed bacteria infected by competent phages. For the subsequent production of recombinant proteins, the heat shock competent *E. coli* (DH5α, Invitrogen/Life Technologies, Carlsbad, CA, USA) were propagated according to standard protocols.

### Phage display library screening

GST-hADAM10(697–748) and GST as a control were covalently bound to magnetic beads following the manufacturer's protocol (Dynabeads M-270 Epoxy, Invitrogen). Beads were first washed three times with 100 µl sodium phosphate buffer. The supernatant was carefully removed and 10 µg of the respective fusion protein were dissolved in 100 µl of sodium phosphate buffer and added to the beads. Following gentle shaking, 50 µl ammonium phosphate buffer were added to the suspension and incubated overnight at 4°C with slow rotation. The protein solution was removed and 200 µl blocking buffer were added. After incubation for 1 h at room temperature with gentle shaking, the beads were washed three times with PBS and 200 µl phage solution were added (100 µl blocking buffer plus 100 µl Phage Display Library Solution (6×10^10^ plaque-forming units (PFU) per ml)). The suspension was incubated at room temperature for 1.5 h. The supernatant was removed and the beads were washed thoroughly (ten times) with wash buffer containing 0.5% (v/v) Tween-20. Elution of the phages was done by adding 150 µl elution buffer and gentle rotation at room temperature for 15 min. The supernatant was transferred and neutralized (1 M Tris, pH 9.0). Phage eluates were serially diluted with sterile LB medium. After 10 min of incubation at 37°C, 500 µl TG1 cells were transduced with 5 µl of the respective dilution, gently mixed and incubated for 30 min at 37°C without shaking. 100 µl of each sample were plated on SOBAG plates containing ampicillin and incubated overnight at 37°C. Transduced colonies were counted in order to determine the phage titer.

### Isolation of phagemid DNA and identification of bound SH3 domains

5 ml LB medium supplemented with ampicillin were inoculated with an *E. coli* clone. Isolation of phagemid DNA was performed using the QIAprep Spin Miniprep Kit (Qiagen, Hilden, Germany) following the manufacturer's protocol. Respective SH3 domain coding regions were sequenced with the BigDye Terminator Kit v1.1 Cycle Sequencing Kit (Applied Biosystems/Life Technologies, Carlsbad, California, USA) using the sequencing primers J-55 ((5′-CCTATTGCCTACGGCAGCC-3′) and H-301 (5′-CAGGGAGTCAAAGGCCGCTTTTGC-3′). Samples were further analyzed by capillary electrophoresis with the Applied Biosystems Genetic Analyzer 3130 and sequence analysis was performed using the VectorNTI 10 software suite (Life Technologies). After translation of the sequences *in silico*, the SH3 domains were identified via BLAST using the Swiss-Prot server. Of note, the phage display screening was performed in two steps, using two separate aliquots of the initially provided library.

### Cell culture and transfection

Peripheral blood mononuclear cells (PBMCs) were isolated by Ficoll density gradient centrifugation from leukocyte concentrates. PHA blasts were generated by stimulating PBMCs with phytohemagglutinin (PHA, Murex Biotech, UK, 0.5 µg/ml) for three to four days. Following Ficoll density gradient centrifugation to remove dead cells, viable PHA blasts were expanded for 10–14 days in RPMI-1640 medium (Gibco, Carlsbad, USA) with 5% (v/v) FCS, antibiotics and 10 U/ml recombinant IL-2 (Chiron, Marburg, Germany). The cell line JFL39.1 was generated from the apoptosis resistant Jurkat variant J16-Rapo by stable transfection with hFasL [32]. In addition, we used the human Jurkat cell line JE6-1 (T cell leukemia, ATCC TIB-152). Both Jurkat cell lines were cultivated in RPMI-1640 medium containing 10% (v/v) FCS and antibiotics. HEK 293T cells (ACC 635, DMSZ) were propagated in DMEM medium (PAA, Pasching, Austria) containing 10% (v/v) FCS, 1% (v/v) L-glutamine (200 mM) and antibiotics. All cells were cultivated in a humidified atmosphere with 5% (v/v) CO_2_ at 37°C.

### Ethics statement

Leukocyte concentrates of healthy blood donors were obtained from the Institute for Transfusion Medicine of the University Hospital Schleswig-Holstein Campus Lübeck. During enrollment, all volunteers have given their written consent that leukocyte concentrates may be used for research purposes. The respective consent forms and the studies on human leukocyte populations were approved by the Ethics Committee of the Medical Faculty of the University of Kiel (file reference: D405/10).

### Verification of selected ADAM10-interacting SH3 domain proteins: cell lysis, pull down analyses, co-immunoprecipitations and Western blotting

For the verification of putative interactors of the cytoplasmic tail of ADAM10, pull down analyses and co-immunoprecipitation experiments were performed from PHA-stimulated PBMCs, Jurkat cells (JE6-1, JFL) or transfected HEK 293T cells. Cells were washed once with ice-cold PBS and lysed in 1% NP-40 lysis buffer containing 5 mM EDTA to prevent autoproteolysis of metalloproteinases and protease and phosphatase inhibitors (sodium orthovanadate, sodium fluoride, sodium pyrophosphate, PMSF, aprotinin, leupeptin, and pepstatin A). Following incubation on ice for 20 min, cell debris was removed by centrifugation. For pull down experiments, lysates were added to glutathione sepharose beads and 10–25 µg of the respective fusion protein. Immunoprecipitations were performed with Protein G sepharose beads (GE Healthcare) and 1–2 µg of the respective antibody. After rotation with the lysate for 2 h at 4°C, beads were washed five times with NP-40 buffer and subjected to electrophoresis and Western blot. Gel electrophoresis and Western blotting were performed following standard protocols using either discontinuous electrophoresis or Bis-Tris NuPAGE gels (Invitrogen). Following protein transfer to nitrocellulose membranes (GE Healthcare), these were blocked with 5% (v/v) BSA or dry milk powder in TBS-T for 1 hour. Membranes were then incubated with primary antibodies diluted in TBS-T for 1 hour at room temperature or overnight at 4°C, washed three times with TBS-T and subsequently incubated for 1 hour with the horseradish peroxidase-conjugated secondary antibody at room temperature. After three additional washes in TBS-T, blots were developed using ECL reagents and films from GE Healthcare.

### Antibodies and expression constructs

The monoclonal antibody clone 11G2, directed against the extracellular part of ADAM10, was obtained from Diaclone (Besancon, France). A polyclonal antiserum against ADAM10 (“animal 1”) was provided by Dr. Paul Saftig (Biochemical Institute, CAU, Kiel). The polyclonal antibody against Grb2 (C-23) was from Santa Cruz Biotechnology (Santa Cruz, CA, USA). Monoclonal antibodies against Lck (clones 4/129, 4/215) were produced in our laboratory after immunization of Balb/c mice with respective GST-tagged SH3 domain fusion proteins [33]. The monoclonal antibody against the myc-tag (46–0603) was purchased from Invitrogen. The rat anti-HA High Affinity monoclonal antibody (3F10) was obtained from Roche (Grenzach-Wyhlen, Germany). The monoclonal anti-Endophilin-A2/EEN antibody (clone 2F5) was from Abcam (Cambridge, UK).

For transient transfection of HEK 293T cells, 4×10^6^ cells were transfected using calcium phosphate-mediated transfection according to standard protocols with plasmids encoding for myc-tagged pombe Cdc15 homology (PCH) protein family members [32]. 18 h post transfection, cells were lysed for pull down and (co-)immunoprecipitation analyses. To confirm the interaction of ADAM10 with members of the sorting nexin family, HA-tagged constructs, kindly provided by S. F. Lichtenthaler (Munich, Germany), were transfected as described [34]. To verify the interaction of ADAM10 with Endophilin-A2/EEN, murine ADAM10 was transfected alone or in combination with a human Endophilin-A2/EEN expression construct (SKU: SC118256) obtained from OriGene Technologies (Rockville, MD, USA). All verification experiments were performed at least three times, mostly using lysates of different origin. Some experiments unfortunately had to be repeated more often because of the variable quality of some of the available antibodies.

## Results and Discussion

Human and murine ADAM10 harbor two putative SH3 domain-binding motifs represented by the amino acid stretches 708–717 (709–718 in mice) (PKLPPPKPLP) and 722–728 (723–729) (RRRPPQP), respectively (Fig. 1). In order to screen for potentially interacting SH3 domains in an unbiased proteome-wide approach, we used the All SH3 Domain Phager from Geneart. The magnetic beads used for selection were coated with GST as a control or with GST-hADAM10(697–748), a GST fusion protein containing the complete intracellular region of human ADAM10. The panning of the library was performed as described [35] with high stringency conditions throughout sample processing to reduce the number of false positive identifications. The screening was done in two separate experiments and revealed that out of the 305 human SH3 domains represented in the used phage library, as many as 38 were precipitated using the GST-hADAM10(697–748) fusion protein (Table 1). It should be noted, however, that most candidates were identified only once (27) or twice (8). The most frequent single hits were found for the SH3 domains of Endophilin-A2 (8 hits), Lck (5) and ZDHHC6 (3). Of note, using this screening procedure, the number of clones does not necessarily mirror the binding specificity of a given fusion protein, because - although the library had been checked for equal representation of individual phage clones according to the manufacturer - there is no guarantee that all SH3 domains are equally abundant in a given experiment. Even though using stringent washing conditions, we still got a relatively high background of GST-bound SH3 domains, which might be explained due to the relative ratio of only 51 amino acids of the intracellular part of ADAM10 compared to 234 amino acids of the GST-tag. SH3 domains which were identified with the GST control or with both GST and GST-hADAM10(697–748) are listed in Table 2. For further analyses, we focused on the SH3 domain proteins that did not precipitate with GST. We do nevertheless not exclude that other candidate binders are among the SH3 domains that precipitated with both GST-hADAM10(697–748) and GST.

**Table 1:** GST-hADAM10(697-748)-precipitated SH3 domains: Function and localization of putative interaction partners according to the UniProt Protein Knowledgebase (UniprotKB).

SH3 domain protein	gene name(s)	Accession number	function	localization	hits
Endophilin-A2	SH3GL1	Q99961	endocytosis, podosome formation	Cyt^1^, PM, Endo, Podo	8
Lck	LCK	P06239	Src-related tyrosine kinase	Cyt, PM, LR	5
ZDHHC6	ZDHHC6	Q9H6R6	palmitoyltransferase	ER	3
Growth factor receptor binding protein 2	GRB2, ASH	P62993	adaptor (growth factor→ Ras)	Cyt, Endo, Nuc, Golgi	2
HS1/HCLS1/LckBP	HCLS1	P14317	adaptor for Lck signaling	PM, Cyt, Mito	2
SH3 domain protein 7, HIP-55	DBNL, SH3P7	Q9UJU6	adaptor, cytoskeleton, endocytosis	Cyt, cell junction, PM, Golgi, ER, Podo	2
Otoraplin	OTOR	Q9NRC9	unknown	secreted	2
Dedicator of cytokinesis protein 4	DOCK4	Q8N1I0	migration, GEF for Rap-1	EMS, Cyt	2
SH3 domain protein 21	SH3D21, C1orf113	A4FU49	unknown	unknown	2
RUN and SH3 domain-containing protein 1	RUSC1	Q9BVN2	adaptor, cytoskeleton	Cyt, Endo, Golgi	2
Rho GEF 38	ARHGEF38	Q9NXL2	GEF for Rho	Cyt	2
PKC and CK substrate in neurons 3	PACSIN3	Q9UKS6	endocytosis	Cyt, PM	1
GRB2-related adaptor protein 2	GRAP2, GADS	O75791	adaptor (LAT, SLP-76)	Cyt, Nuc, Endo	1
c-Src	Src	P12931	tyrosine kinase	Cyt, PM, Nuc, Mito	1
c-Abl	ABL1	P00519	tyrosine kinase, cytoskeleton	Cyt, Nuc, Mito	1
Sorting nexin 18	SNX18	Q96RF0	endocytosis, vesicle transport	PM, Endo, Cyt	1
Adaptor protein crk (c-Crk)	Crk	P46108	adaptor, actin cytoskeleton	Cyt, PM	1
Rho GEF 19	ARHGEF19	Q8IW93	GEF for RhoA, cytoskeleton	Cyt	1
Peroxin-13	PEX13	Q92968	peroxisomal import	Peroxisome membrane	1
Ephexin-1	NGEF	Q8N5V2	GEF for RhoA, Rac1, CDC42	Cyt, PM, growth cone	1
Rho GAP 32, RICS protein	ARHGAP32	A7KAX9	GAP for RhoA, Rac1, CDC42	Cyt, cell junction, PM, Golgi, ER, Endo	1
ARHGEF16, Ephexin-4	ARHGEF16	Q5VV41	GEF for RhoG	Cyt	1
Growth arrest-specific protein 7	GAS7	O60861	neuronal differentiation	Cyt, ruffles	1
unconventional myosin 1-E	MYO1E	Q12965	cytoskeleton	Cyt, cell junction	1
Rho GEF 4	ARHGEF4	Q9NR80	GEF for RhoA, Rac1, CDC42	Cyt, PM, ruffles	1
Vinexin SH3 #2	SORBS3, SCAM1	O60504	cytoskeleton	Cyt, cell junction, Nuc	1
Vinexin SH3 #3	SORBS3, SCAM1	O60504	cytoskeleton	Cyt, cell junction, Nuc	1
RIMS Binding protein 3A	RIMBP3A	Q9UFD9	unknown	unknown	1
Rho GAP 33, Sorting nexin 26	ARHGAP33	O14559	protein transport	Cyt, PM	1
LIM and SH3 domain protein 1	LASP1	Q14847	adhesion, cytoskeleton	Cyt	1
unconventional myosin VIIa	MYO7A	Q13402	cytoskeleton, intracellular movement	Cyt	1
Disks large homolog 1	DLG1	Q12959	scaffold protein	ER, PM, cell junction	1
Erythroid α spectrin	SPTA1	P02549	cell shape, actin cytoskeleton	Cyt	1
non erythroid α spectrin (fodrin)	SPTAN1	Q13813	Ca^2+^-dependent movement	Cyt	1
SH3 domain-containing protein 19, Eve-1	SH3D19	Q5HYK7	cell morphology, cytoskeleton	Cyt, Nuc	1
RIMS Binding protein 2	RIMB2	O15034	synaptic transmission	PM, cell junction	1
peripheral PM protein CASK	CASK	O14936	calmodulin-dependent serine kinase	Nuc, Cyt, PM (binds APP, syndecan)	1
Dedicator of cytokinesis protein 2	DOCK2	Q92608	migration, GEF for Rac1,Rac2	EMS, PM, Cyt	1

1 **Abbreviations**: Cyt, cytosol; EMS, endomembrane system; ER, endoplasmic reticulum; Endo, endosomes; LR, lipid rafts; Mito, Mitochondria; Nuc, Nucleus; PM, plasma membrane; Podo, podosomes.

**Table 2:** GST/GST-hADAM10(697–748)- and GST-interacting SH3 domains.

SH3 domain (gene name)	Accession number	hADAM10 (697–748) (n = 216)^1^	GST (n = 75)^1^
ARHG9	O43307	72	27
IASPP	Q8WUF5	21	5
NOXO1	Q8NFA2	10	2
Eps8R1	Q8TE68	7	1
ZO1	Q07157	6	2
SH3TC2	Q8TF17	4	3
SH3BP4	Q9P0V3	4	2
NCF1	P14598	4	1
SKAP1/55R	Q86WV1	4	1
MYO15A	Q9UKN7	3	1
DLG5	Q8TDM6	3	1
SH3RF3	Q8TEJ3	3	1
SH3PXD2B	A1X283	2	2
SHANK1	Q9Y566	2	1
CACNB4	O00305	2	1
ARHGAP4	P98171	2	1
Eps8R3	Q8TE67	1	3
CACB3	P54284	1	2
MYO7B	Q6PIF6	1	1
ASPP2	Q13625	1	1
SHANK2	Q9UPX8	1	1
MYO15B	Q96JP2	1	1
MACC1	Q6ZN28	1	1
DOCK3	Q8IZD9	1	1
NOXA1	Q86UR1	-	2
MPP2	Q14168	-	2
FCSD1	Q86WN1	-	1
NPHP1	O15259	-	1
SKAP2	O75563	-	1
CSKI1	Q8WXD9	-	1
DLG3	Q92796	-	1
TXK	P42681	-	1
DNMBP	Q6XZF7	-	1
MATK	P42679	-	1

1 number of sequenced phage clones precipitated with GST-hADAM10(697–748) and GST, respectively.

The identified putative SH3 domain-containing interactors of the intracellular domain of ADAM10 belong to different protein families of adaptor proteins, non-receptor tyrosine kinases or palmitoyltransferases. As listed in Table 1, many of the candidate adaptor proteins are involved in membrane shaping and trafficking (e.g. endophilins, sorting nexins (SNXs), PCH proteins) or mediate cytosolic protein-protein interactions that are somehow linked to cytoskeletal reorganization. Importantly, several of the candidate interactors or related proteins have been identified as binding partners for other ADAM family proteases including ADAM9, 12, 13 or 15 (see below).

Before we started co-precipitation or pull down experiments to investigate individual interactions, we verified the presence of selected candidate proteins in PHA blasts, cloned T cells and Jurkat cells by Western blot. Moreover, we performed a comprehensive pull down screening from PHA blasts or Jurkat cells to get additional information about possible interactions with SH3 domains and/or full length proteins (supplementary Fig. S1–S5). Being aware that functional data on the relevance of individual interactions still have to be established, along with the verification of ADAM10 binding, we discuss the potential role of selected binding partners for ADAM10 biology and provide a hypothetical model for their role in ADAM10 biology (Fig. S6).

### Endophilins

During our screening, the SH3 domain of Endophilin-A2 (SH3GL1, EEN) was found eight times in GST-hADAM10(697–748) precipitates but never with GST alone. Endophilins (encoded by the EEN gene family) are cytosolic proteins with an N-terminal Bin-Amphiphysin-Rvs (N-BAR) domain and a C-terminal SH3 domain. In general, endophilins contribute to the formation of membrane tubules during endocytosis by inserting into the membrane and inducing/changing membrane curvature [23], [36]–[38]. Whereas Endophilin-A2 is ubiquitously expressed, Endophilin-A1 is prominently found in the brain and Endophilin-A3 mainly in testis, but also in liver and brain [37]. Notably, the close relative Endophilin-A1 was identified in an earlier study as an interaction partner for the precursor but not the processed forms of ADAM9 and ADAM15 in a yeast two-hybrid screen [22]. These interactions were confirmed using fusion proteins and co-precipitations from eukaryotic cells overexpressing both binding partners. It was proposed that Endophilin-A1 may have a role in regulating the function of ADAM9 and ADAM15 by modulating their intracellular processing, transport, or final subcellular localization. Like the other family members, Endophilin-A2 interacts with dynamin and synaptojanin via its C-terminal SH3 domain and is involved in endocytotic processes [37]. Furthermore, the SH3 domain of Endophilin-A2 was shown to bind the proline-rich region of the Rho GTPase-activating protein (RhoGAP) BPGAP1, leading to EGF-stimulated endocytosis of the EGF receptor and ERK1/2 phosphorylation [39]. Notably, due to the spatial aggregation of several endophilins at sites of endocytosis, an individual SH3 interaction (e.g. to dynamin) of one endophilin does not rule out that the neighboring molecule binds to the PRR of another protein (e.g. ADAM10).

**Figure 2:**
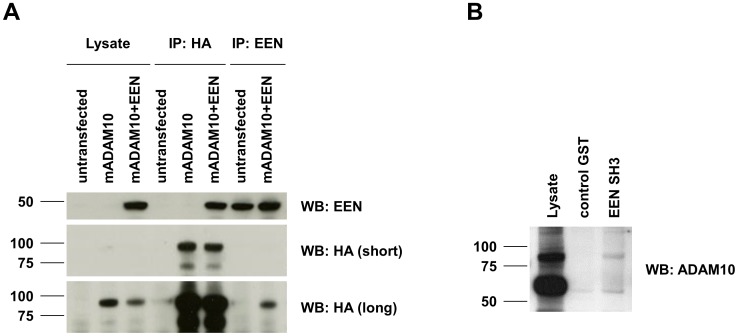
Verification of the interaction between ADAM10 and EEN. (**A**) In order to verify the potential interaction of ADAM10 with Endophilin-A2/EEN, HEK 293T cells were either left untransfected or transfected with HA-tagged murine ADAM10 alone or in combination with human Endophilin-A2/EEN. 18 h later, the cells were lysed and immunoprecipitations (IPs) were performed with monoclonal antibodies directed against the HA-tag (clone 3F10) or EEN (clone 2F5), respectively. Protein input for IPs was 1.8 or 2 mg of protein, respectively. Of note: at the employed exposure time, endogenous EEN is hardly detectable in the whole cell lysates containing a total of 10 µg of protein. (**B**) Pull down analyses were performed from PHA blasts (day 16) using a GST fusion protein containing the SH3 domain of EEN coupled to GST (EEN SH3) and GST as a control. The subsequent Western blot was probed with anti-ADAM10 (clone 11G2).

In order to verify the interaction of ADAM10 with Endophilin-A2/EEN, murine HA-tagged ADAM10 and human Endophilin-A2/EEN were transiently overexpressed in HEK 293T cells (either alone or in combination). As mentioned, the intracellular domains of murine and human ADAM10 differ only in one amino acid outside the PRDs (Fig. 1). 18 hours post transfection, the cells were lysed and precipitations were performed using antibodies directed against HA or Endophilin-A2/EEN. Precipitating HA-tagged ADAM10 led to co-precipitation of EEN (Fig. 2A, upper panel). Moreover, immunoprecipitation of EEN resulted in co-precipitated ADAM10, here detected with the anti-HA antibody 3F10 (Fig. 2A, lower panels). Of note, the anti-Endophilin-A2/EEN monoclonal antibody 2F5 efficiently precipitated endogenous EEN from untransfected cells (protein input ∼2 mg per IP). At the given short exposure time, endogenous EEN was not detected in whole cell lysates (total protein input: 10 µg). The ADAM10/EEN interaction was also investigated by pull down experiments using the GST-tagged SH3 domain of EEN (EEN SH3) for precipitation of endogenous ADAM10 from lysates of day 16 PHA blasts (Fig. 2B). The immature form of ADAM10 (proADAM10, 82 kDa) was apparently precipitated more effectively with the SH3 domain of EEN. Using mAb 11G2 for Western blot detection of ADAM10, we did not detect equal amounts of mature ADAM10 in EEN SH3 precipitates although this form was highly abundant in the cellular lysate. Similar results were obtained in the pull down screening (Fig. S2). In EEN SH3 precipitates, amongst other bands, the polyclonal anti-ADAM10 antiserum stained a protein band migrating at the height of the ADAM10 pro-form in the corresponding immunoprecipitate. Thus, it might be interesting in this context to further address the issue of whether individual SH3 domain proteins preferentially associate with the pro-form, the mature form or even the intracellular domain of ADAM10 as it has been suggested for the interaction of endophilins with ADAM9 and ADAM15.

### Non-receptor protein tyrosine kinases

SH3 domains of several non-receptor protein tyrosine kinases (PTKs) including Src family members and Abl were also identified as putative interactors of ADAM10 in the phage screen. The Src family of cytosolic non-receptor protein tyrosine kinases consists of nine members (Blk, Fgr, Frk, Fyn, Hck, Lck, Lyn, Src and Yes) (see [40] for a recent review). Src kinases initiate and direct numerous signal transduction processes, including for example antigen receptor-driven activation of T and B cells. The related Abl tyrosine kinase has been implicated in cell migration, cell adhesion and apoptosis. Moreover, Abl contributes to TCR signaling since it is phosphorylated and activated by Src kinases and in turn phosphorylates receptor tyrosine kinases, e.g. the epidermal growth factor receptor (EGFR) to modulate receptor endocytosis [41], [42]. Interactions with non-receptor PTKs have been previously reported for several ADAM family members. It was shown that the SH3 domain of Src interacts with the cytoplasmic domain of ADAM12 [12], [13] and that ADAM12 gets phosphorylated by v-Src [13]. Later, Poghosyan and colleagues demonstrated that the cytoplasmic tail of ADAM15 binds to the SH3 domains of Src, Lck, Fyn and Abl [10]. Moreover, PKC activation induced an increased phosphorylation of ADAM15 and enhanced its association with non-receptor PTKs whereas dephosphorylation led to reduced binding of ADAM15 to Hck, Lck, Fyn and the adaptor protein Grb2. In addition, Hck and Lck were able to phosphorylate ADAM15 *in vitro* [10]. Notably, the interactions of ADAM15 with Src kinases and Abl were also found using a similar phage display approach [43].

**Figure 3:**
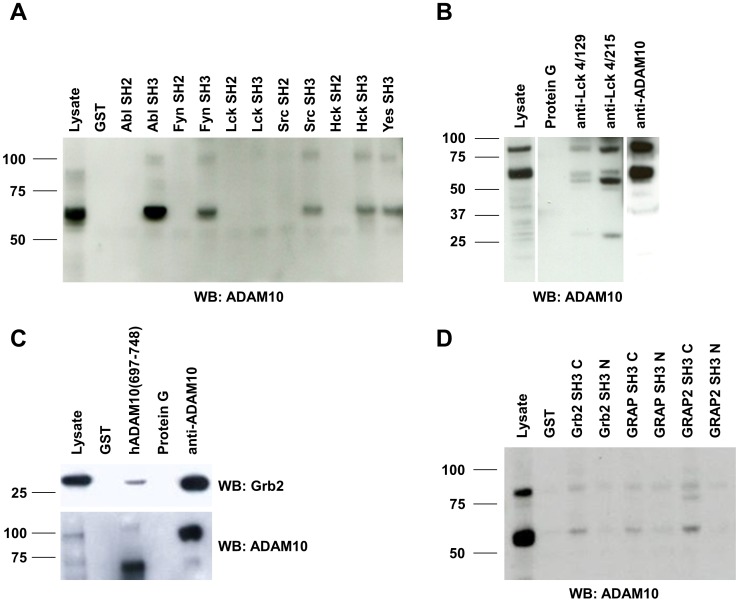
Interactions between ADAM10 and non-receptor protein tyrosine kinases and adaptor proteins of the Grb2 family. (**A**) Lysates from Jurkat T cells (here JFL) were used for pull down analyses with SH2 or SH3 domain fusion proteins of non-receptor PTKs as indicated. GST served as a negative control. Protein input of the whole cell lysate was 15 µg. 10 µg of the respective fusion proteins were used for precipitation from 1 ml of cell lysate with 3.2 mg/ml of protein. MAb 11G2 was used to detect ADAM10 after Western blotting. (**B**) Immunoprecipitations were performed from Jurkat T cells (here JE6-1) using 2 µg of mAbs against Lck (clones 4/129 and 4/215) or ADAM10 (clone 11G2). Protein G beads served as a control for unspecific binding. Input of the cellular lysate was 15 µg; precipitates were performed from 1 ml of lysate (1 mg/ml protein). MAb 11G2 was used to detect ADAM10 by Western blotting. (**C**) Jurkat cells (JE6-1) were lysed and one ml of lysate containing 900 µg/ml protein was subjected to precipitation using 10 µg GST or the GST fusion protein containing the intracellular part of human ADAM10 (hADAM10(697–748)). In parallel, ADAM10 was precipitated using 2 µg/ml of mAb 11G2 with protein G beads serving as a control. Precipitated proteins were separated by SDS-PAGE and blotted on nitrocellulose. The blot was stained with a polyclonal anti-Grb2 antibody and re-probed with a polyclonal anti-ADAM10 antiserum (“animal 1”). (**D**) C- and N-terminal SH3 domains of Grb2, GRAP and GRAP2 fused to GST (10 µg each) were used for precipitations from Jurkat T cells (here JFL; 2.2 mg/ml protein input per precipitation) with GST alone serving as a control. 15 µg protein of the whole cell lysate were included as a reference. ADAM10 was detected with mAb 11G2.

Screening for ADAM10-interacting SH3 domains, we identified several domains of non-receptor PTKs that precipitated with hADAM10(697–748)-coated beads. Lck was found most frequently, but also SH3 domains of Src and Abl were identified as putative interaction partners. In order to verify these interactions, we performed pull down experiments with lysates of FasL-transfected (JFL) Jurkat cells using recombinant SH3 domains of several candidate kinases fused to GST and GST and/or respective SH2 domains as controls (Fig. 3A). Western blots were stained for ADAM10 using mAb 11G2 which seems to predominantly stain the mature form of the protease in most cases. As depicted in Fig. 3A, endogenous mature ADAM10 could be readily precipitated with 10 µg of GST-coupled SH3 domains of Abl, Fyn, Src, Hck and Yes but not with GST alone or the SH2 domains of the respective kinases. Surprisingly, the precipitation of ADAM10 with the SH3 domain of Lck was less efficient in this experiment. However, at longer exposure times, ADAM10 became also visible in precipitates with SH3 domains of Lck (data not shown). In addition, repeating the pull down experiments with different preparations and higher amounts (25 µg) of the fusion proteins confirmed the Lck interaction (supplementary Fig. S1). Under these conditions, SH3 domains of different Src-related kinases (Fig. S1), of Abl and of the Tec-kinase Itk (Fig. S4) apparently preferentially precipitated different forms of ADAM10 from PHA blasts or Jurkat cells as stained with the polyclonal anti-ADAM10 antiserum. It should be mentioned in this context that neither GST nor any of the used SH2 domains precipitated proteins detected by this anti-ADAM10 antiserum. As an additional proof of the Lck/ADAM10 interaction, we performed precipitations from Jurkat cells (JE6-1) using two monoclonal antibodies that we had generated against Lck and co-precipitated mature (and pro-) ADAM10 as visualized with mAb 11G2 on the Western blot (Fig. 3B). As a positive control, we included an ADAM10 IP using the mAb 11G2.

### The palmitoyltransferase ZDHHC6

The third most frequently identified SH3 domain was annotated in the library data sheet as ‘homology to Ablphilin-2’ and might be better known as ‘zinc finger DHHC domain-containing protein 6’ (ZDHHC6). ZDHHC6 belongs to the DHHC family of palmitoyltransferases. Unfortunately, at present there are hardly any reagents available to address the potential interaction of ZDHHC6 with ADAM10. In general, transfer of palmitoyl groups increases protein hydrophobicity favoring membrane association. As a reversible posttranslational modification, palmitoylation contributes to the intracellular trafficking of proteins between membrane-shielded compartments and thereby determines their intracellular localization and contributes to protein clustering in platforms such as lipid rafts. As an example, calnexin, a major ER chaperone involved in glycoprotein folding, is modified by the ER-resident palmitoyltransferase DHHC6 leading to a preferential localization of calnexin to the perinuclear rough ER [44]. Moreover, Ablphilin-2 (ZDHHC16, Aph2) was shown to interact with ER-associated c-Abl and to promote ER-stress and apoptosis [45]. The available information about other DHHC proteins including ZDHHC6, however, is very limited. Regarding ADAM family proteases, palmitoylation as a posttranslational modification has not been described so far. However, substrates of ADAM10 and ADAM17 are known to be palmitoylated, leading to altered functionality and localization. For example, palmitoylation of the Fas ligand (FasL, CD95L) is essential for its killing capacity and also for its proteolytic processing by ADAM10 and SPPL2a [46]. In contrast, palmitoylation of TNF-α is involved in lipid raft positioning of the cytokine and seems to interfere with the cleavage/degradation of TNF intracellular fragments, but is not involved in the regulation of its ectodomain shedding by ADAM17 [47]. Also, the shedding of the NKG2D ligands MICA/B (MHC-class-I-related chain A/B) was shown to be regulated by their localization in cholesterol-enriched domains, while inhibition of palmitoylation by 2-bromopalmitate led to reduced shedding [48], [49]. With regard to regulation of ADAM10, it was reported that only palmitoylated Tetraspanin12 might serve as a binding partner for ADAM10 to promote its localization and maturation and thereby for instance facilitating the ADAM10-dependent proteolysis of amyloid precursor protein [50]. Thus, ZDHHC6 could not only modulate the palmitoylation status of ADAM10, but also of certain substrates to alter their localization, accessibility or processing and degradation for example in tetraspanin or lipid raft platforms. These hypotheses could be addressed in future experiments as soon as respective reagents become available.

### Grb2 family adaptor proteins

Adaptor proteins play a fundamental role in orchestrating cellular signaling processes. They are composed of different modules that either form interaction domains or binding sites for constitutive or activation-dependent protein-protein interactions [51]. The Grb2 family belongs to the cytosolic adaptor proteins and consists of three members: the growth factor receptor-bound protein 2 (Grb2) is widely expressed in different cell types and tissues, whereas the Grb2-related adaptor protein (GRAP) and Grb2-related adaptor protein 2 (GRAP2/MONA/GADS) are more restricted to hematopoietic cells. All three proteins consist of one SH2 domain flanked by two SH3 domains. This allows simultaneous interactions with several proteins and the formation of molecular networks [52].

In our screen, we found that SH3 domains of Grb2 and GRAP2 precipitated with the intracellular portion of ADAM10. To verify the putative interaction of ADAM10 with Grb2, Jurkat T cells (JE6-1) were lysed and a pull down using the GST-hADAM10(697–748) fusion protein and an immunoprecipitation with mAb 11G2 were performed as depicted in Fig. 3C. Western blotting using a polyclonal anti-Grb2 antibody (C-23) revealed that Grb2 was precipitated with the GST fusion protein containing the intracellular domain of ADAM10, but not with GST. Furthermore, Grb2 was effectively co-precipitated with ADAM10 (Fig. 3C). Note that the blot was reprobed for ADAM10 using the polyclonal antiserum (“animal 1”). In order to assess the binding of other members of the protein family to ADAM10, we used GST fusion proteins comprising the C- or N-terminal SH3 domains of Grb2, GRAP and GRAP2. Interestingly, as shown for other interaction partners before [53], we found differential binding of ADAM10 to the C- or N-terminal SH3 domains of all three adaptor proteins. Using mAb 11G2, ADAM10 was more prominently detected in the presence of C-terminal SH3 domains, whereas less protein was observed when precipitated with the N-terminal SH3 domains (Fig. 3D). Similar differences were also seen in the pull down screen (supplementary Fig. S2). However, although the polyclonal anti-ADAM10 antiserum used for staining of ADAM10 detected only two bands in ADAM10 IPs performed with mAb 11G2, the band patterns stained in SH3 domain precipitates formed by Grb2, GRAP and GRAP2 were quite heterogeneous.

Notably, also for Grb2 family members, interactions with several ADAM family members were previously described. For example, it was shown that immunoprecipitation of endogenous Grb2 led to co-precipitation of ADAM12 [13]. Moreover, internalization of ADAM12 was found to be dependent on Grb2 and knockdown of endogenous Grb2 resulted in reduced internalization [21]. As mentioned before, ADAM15 also binds to Grb2 and dephosphorylation of cellular extracts decreased this interaction, possibly arguing for a cooperative SH2/SH3 domain interaction network [10].

### Sorting nexins

The sorting nexins (SNXs) form a large family of ubiquitously expressed proteins which also function as regulators of intracellular trafficking, endocytosis and signal transduction. SNX9, SNX18 and SNX33 constitute a subfamily since they contain SH3 domains and display a high overall homology. Similar to endophilins, sorting nexins contain an N-BAR domain and are thus implicated in the modulation of membrane curvature and tubulation in endosomal sorting or, more general, in endo- and exocytosis [24], [25]. As examples, SNX9 promotes the internalization of the TNF receptor (TNFR) and influences the degradation of the EGF receptor (EGFR) after EGF signaling [54]. Furthermore, SNX9 participates in the reorganization of the actin cytoskeleton by promoting the activation of the Arp2/3 complex [55].

**Figure 4:**
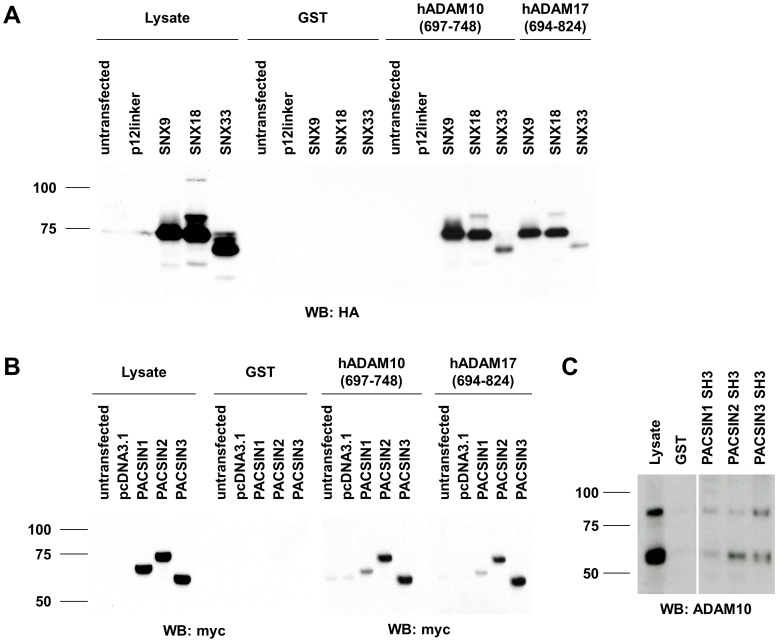
The intracellular domains of ADAM10 and ADAM17 interact with sorting nexins and PACSINs. (**A**) HEK 293T cells were either left untransfected or were transfected with a control vector (p12linker) or with HA-tagged SNX9, SNX18 or SNX33. 18 h later, cells were lysed and precipitations were performed with 25 µg of GST, GST-hADAM10(697–748) or GST-hADAM17(694–824), respectively. Western blots were developed using mAb 3F10 directed against the HA-tag of the sorting nexins. Lysates (20 µg/lane) were stained as a control. (**B**) HEK 293T cells were left untransfected or transfected with control vector (pcDNA3.1) or with myc-tagged PACSIN1, PACSIN2 or PACSIN3. 18 h post transfection, cells were lysed and precipitations were performed with 10 µg of GST, GST-hADAM10(697–748) or GST-hADAM17(694–824). Precipitated proteins were separated by SDS-PAGE and transferred to nitrocellulose membranes. Blots were developed with mAb clone 46–0603 directed against the myc-tag. Of the whole cell lysates, 5 µg of protein were separated to verify efficient transfection. (**C**) Recombinant GST fusion proteins (15 µg) containing individual SH3 domains of PACSIN1-3 were used to precipitate endogenous ADAM10 from lysates of Jurkat T cells (JFL, 1 ml each; 2.2 mg/ml). GST served as a negative control. 10 µg of whole cell lysate was used as a control to detect ADAM10 using mAb 11G2.

SNX18 was identified as a putative SH3 domain binding partner for ADAM10. In subsequent experiments, we found that ADAM10 interacts with exogenously expressed SNX9, SNX18 and SNX33. To address this, HEK 293T cells were transiently transfected with HA-tagged sorting nexin constructs and precipitates using GST or the GST-hADAM10(697–748) fusion protein were formed and analyzed. As shown in Fig. 4A, SNX9, SNX18 and SNX33 were readily precipitated as revealed by Western blot using an anti-HA antibody (clone 3F10). Here, the strongest interaction was observed with SNX9 and SNX18 (Fig. 4A). In this context, we additionally analyzed whether the SNXs also interact with ADAM17 employing a hADAM17(694–824) fusion protein containing the putative SH3 binding motif PAPQTPGR (amino acids 731–738) [56]. In case of ADAM17, strongest binding was again seen with SNX9 and SNX18 (Fig. 4A). Not unexpectedly, sorting nexins (and especially SNX9) had also been identified as interaction partners of other ADAM proteases (including ADAM9 and ADAM15). Howard and colleagues demonstrated binding of SNX9 to the precursor forms of ADAM9 and ADAM15, but not to the mature form, proposing a potential role of SNX9 in regulating processing, function and localization of the metalloproteases [22]. ADAM15 was also reported to interact with SNX33 in a phage display screen [43], showing preferential binding to specific isoforms of ADAM15 containing the C-terminal proline-rich region [57].

### PACSINs

The SH3 domain of PACSIN3 (PKC and casein kinase substrate in neurons 3) was identified as a putative interactor for ADAM10. PACSIN3 and its close relatives PACSIN1 and −2 belong to the pombe Cdc15 homology (PCH) protein family, a family of adaptor proteins involved in cytoskeleton-to-membrane crosstalk. PCH proteins share a Fes-CIP4 homology (FCH) domain within their F-BAR domain. Like other proteins with BAR domains, PCH proteins change membrane curvature and are involved in the regulation of endo- and exocytosis and vesicular transport [58], [59]. Whereas PACSIN1 is mainly expressed in neurons, PACSIN2 and PACSIN3 seem to be broadly distributed [60]–[63]. Moreover, most PCH proteins contain functional SH3 domains at the C-terminus. Also PACSIN proteins were already shown to interact with cytosolic portions of other ADAM proteins. As mentioned, Cousin and colleagues were the first to describe a SH3 domain-mediated interaction of PACSIN2 and ADAM13 in *Xenopus* embryos and demonstrated that overexpression of X-PACSIN2 could rescue developmental alterations induced by overexpression of ADAM13 [11]. PACSIN3 was identified as an interactor of ADAM12 in a yeast two-hybrid screen and this interaction was further substantiated by pull down and co-immunoprecipitation analyses. Overexpression of PACSIN3 resulted in enhanced shedding of the ADAM12 substrate heparin-binding EGF-like growth factor (proHB-EGF) upon stimulation, whereas knockdown reduced proteolysis [15].

Since PACSIN3 was already on the list of ADAM interactors, we followed this in more detail. When we transiently overexpressed myc-tagged PACSINs in HEK 293T cells, we could readily precipitate all PACSINs with the GST-hADAM10(697–748) fusion protein but not with a GST control (Fig. 4B). Interestingly, the association with PACSIN3 was more pronounced compared to PACSIN1 and PACSIN2. Moreover, GST fusion proteins containing the individual SH3 domains of the three different PACSINs precipitated mature as well as immature ADAM10 (Fig. 4C). The ADAM10 interaction was also seen for all three PACSINs in the complementary pull down screen (supplementary Fig. S3). In parallel, we investigated a potential binding of PACSINs to ADAM17, using the GST-hADAM17(694–824) fusion protein. All PACSINs were efficiently precipitated, but the strongest binding was again observed for PACSIN3 (Fig. 4B).

### Drawbacks and other candidate interactors

As detailed above, we tried to verify as many interactions as possible using a variety of different approaches. However, co-localization studies of endogenous (and also of overexpressed) proteins (e.g. by confocal imaging) turned out to be undoable with available anti-human ADAM10 antibodies, although ADAM10 is expressed at high levels on T lymphocyte populations [6]. Also, co-immunoprecipitation of endogenous proteins proved to be difficult and Western blotting using different electrophoresis systems in combination with either monoclonal antibodies or polyclonal antisera were not always superimposable. In some cases, interactions could only be verified by overexpression and co-immunoprecipitation or pull down of tagged full-length proteins using adequate controls. We therefore tested a series of GST fusion proteins containing isolated SH3 domains, full length proteins or SH2 domains (as controls) of candidate interactors in pull down assays from T cell blasts or Jurkat cells to complement the data obtained by screening the phage library. As mentioned, most interactions that were identified based on the phage display screen were also seen in pull down analyses. Although the band patterns obtained after staining of Western blots with the polyclonal anti-ADAM10 antiserum were occasionally somewhat confusing, from this pull down experiments a few other interactors might also be worth testing in future experiments. These include the adaptor protein Nck (Fig. S3) which seems to interact with ADAM10 with at least one of its three SH3 domains, the Tec-kinase Itk mentioned before, the p85 subunit of PI3 kinase, NCF1 (p47phox, the 47-kilodalton cytosolic subunit of the NADPH oxidase complex), and CD2BP1, a PACSIN-related member of the PCH protein family (Fig. S4).

### Shared interaction partners: ADAM10 and FasL

Interestingly, over the last decade, we and others have shown that the ADAM10 substrate FasL [4]–[6] interacts with a very similar subset of SH3 domain proteins as now shown for ADAM10. For example, FasL binds to non-receptor PTKs (Fgr, Fyn, Lyn, Src, Tec and Yes), PI3 kinase, Nck, Grb2-related proteins, NCF1 [30], [35], [53], [64], sorting nexins [35], [65] and PCH proteins [32], [53], [65], [66]. The functional implication of this surprising coincidence is presently not understood. In the case of FasL, several interactors were meanwhile shown to regulate its intracellular storage and its mobilization to the cell surface [32], [66]–[69]. Although FasL and ADAM10 are quite different regarding their intracellular trafficking and membrane appearance, it seems obvious that molecules regulating membrane-to-cytoskeleton-signaling are coupled to both proteins. Moreover, we also observed a differential interaction of SNXs and PCH proteins with the full length form of the protein or its N-terminal fragments generated by ADAM10-mediated proteolysis [34]. Since ADAM10 itself is subject to proteolytic cleavage by ADAM9, ADAM15 and γ-secretase [70], it might be also possible that SH3 domain proteins differentially bind to the full length protease (i.e. to the pro- or mature form) or to its processed fragments (Fig. 1B and supplementary Fig. S6).

Provided that reliable tools for the intracellular or biochemical detection of human ADAM10 (and/or ADAM17) become available, based on the present findings, the functional relevance of individual interactions for the regulation of ADAM10 expression and/or activity could be addressed using similar approaches that have been successfully used for the characterization of SH3 domain proteins in the context of other ADAM proteases [20], [21], [71]. Such studies could be complemented using inhibitors (e.g. for Src kinases) and knockdown approaches targeting potential interactors by siRNA or expressing mutants that prevent SH3 binding. A hypothetical scenario for the role of individual SH3 domain interactors in the regulation of ADAM10 biology is depicted in Fig. S6. As discussed before, it seems likely that individual interactors that turned out to have regulatory functions in the context of related proteases also modulate ADAM10 function. The different protein families that comprise the pool of binding partners suggest a role in ADAM10 storage or transport in membrane positioning (e.g. in plasma membrane platforms), in substrate association, in enzymatic activity, in recycling, or in translocation and/or degradation of proteolytically processed ADAM10. Undoubtedly, the knowledge about the role of individual intracellular interactors for the regulation of ADAM10 expression and activity would also foster further approaches to target the protease for therapeutic intervention [72].

## Conclusions

Using a phage display library screen, we identified an array of individual SH3 domains as putative interaction sites for the PRRs of ADAM10. The protein families that comprise these SH3 domains include non-receptor tyrosine kinases and adaptor proteins that mostly regulate endo- and exocytosis, membrane trafficking, protein positioning and/or membrane-to-cytoskeleton interactions. Based on the initial biochemically verification of different putative interaction partners and based on the overlap with reported functionally relevant interactions with other ADAM proteases, we consider these proteins promising targets for further analyses to address whether SH3 domain proteins that bind to the two intracellular PRRs of ADAM10 also play a role for the subcellular localization and activity of the protease.

## Supporting Information

Figure S1 **Immunoprecipitation and pull down from human PHA blasts – Src related kinases.** PHA-stimulated T cells were lysed in NP40 lysis buffer containing EDTA and protease and phosphatase inhibitors. Immunoprecipitations were performed from 1 ml of cell lysate (equivalent to 50×10^6^ cells) using 2 µg/ml of the indicated anti-ADAM10 or anti-ADAM17 antibodies. Precipitations with GST as a control or GST fusion proteins containing SH2 and/or SH3 domains of the Src-related kinases Fyn, Lck, Src, Hck and Yes were done using 25 µg/ml lysate of the respective fusion proteins. (A) Ponceau S staining following Western transfer. (B) ADAM10 immunoblot using the polyclonal anti-ADAM10 antibody (“animal 1”) - short exposure time. (C) ADAM10 immunoblot using the polyclonal anti-ADAM10 antibody (“animal 1”) - long exposure time.
(TIF)

Figure S2 **Immunoprecipitation and pull down from human PHA blasts – Grb-2 related adaptor proteins, EEN and FBP17.** PHA-stimulated T cells were lysed in NP40 lysis buffer containing EDTA and protease and phosphatase inhibitors. Immunoprecipitations were performed from 1 ml cell lysate (equivalent to 50×10^6^ cells) using 2 µg/ml of the indicated anti-ADAM10 or anti-ADAM17 antibodies. Precipitations with GST as a control or GST fusion proteins containing SH2 and/or SH3 domains or full length proteins of Grb2, GRAP, GRAP2, EEN and FBP17 were done using 25 µg/ml lysate of the respective fusion proteins. (A) Ponceau S staining following Western transfer. (B) ADAM10 immunoblot using the polyclonal anti-ADAM10 antibody (“animal 1”) - short exposure time. (C) ADAM10 immunoblot using the polyclonal anti-ADAM10 antibody (“animal 1”) - long exposure time.
(TIF)

Figure S3 **Immunoprecipitation and pull down from human Jurkat cells (JE6-1) – PACSINs and Nck1.** Jurkat cells were lysed in NP40 lysis buffer containing EDTA and protease and phosphatase inhibitors. Immunoprecipitations were performed from 1 ml cell lysate (equivalent to 50×10^6^ cells) using 2 µg/ml of the indicated anti-ADAM10 or anti-ADAM17 antibodies. Precipitations with GST as a control or GST fusion proteins containing SH2 and/or SH3 domains or full length proteins of PACSINs or Nck1 were done using 25 µg/ml lysate of the respective fusion proteins. (A) Ponceau S staining following Western transfer. (B) ADAM10 immunoblot using the polyclonal anti-ADAM10 antibody (“animal 1”) - short exposure time. (C) ADAM10 immunoblot using the polyclonal anti-ADAM10 antibody (“animal 1”) - long exposure time.
(TIF)

Figure S4 **Immunoprecipitation and pull down from human Jurkat cells (JE6-1) – Abl, Itk, PI 3K, phox47 and CD2BP1.** Jurkat cells were lysed in NP40 lysis buffer containing EDTA and protease and phosphatase inhibitors. Immunoprecipitations were performed from 1 ml cell lysate (equivalent to 50×10^6^ cells) using 2 µg/ml of the indicated anti-ADAM10 or anti-ADAM17 antibodies. Precipitations with GST as a control or GST fusion proteins containing SH2 and/or SH3 domains or full length proteins of Abl, Itk, PI 3K, phox47 or CD2BP1 were done using 25 µg/ml lysate of the respective fusion proteins. (A) Ponceau S staining following Western transfer. (B) ADAM10 immunoblot using the polyclonal anti-ADAM10 antibody (“animal 1”) - short exposure time. (C) ADAM10 immunoblot using the polyclonal anti-ADAM10 antibody (“animal 1”) - long exposure time.
(TIF)

Figure S5 **Coomassie blue staining of fusion proteins used for pull down experiments.** (A-C) Prior to the precipitations from cell lysates as depicted in the supplementary Figures S1–S4, 25 µg of all fusion proteins were checked after separation by SDS-PAGE for degradation by in-gel-staining with coomassie blue. (D–E) Potentially degraded (“old”) fusion proteins were compared to freshly thawed material (“fresh”) and replaced if necessary.
(TIF)

Figure S6 **The complex ADAM biology – a hypothetical model depicting putative regulation by SH3 domain proteins.** ADAM proteases are synthesized in the rough endoplasmatic reticulum (ER) with a signal peptide that is removed when entering the trans-Golgi network (TGN). It is believed that the pro-domain is cleaved off in the Golgi compartment by protein convertases such as furin. In certain cell types, ADAM10 activity has been associated with a lysosomal compartment. It is, however, not clear whether this organelle association is involved in storage or translocation of the protease or whether the protease exerts intracellular activity in an endo-lysosomal compartment. When ADAM10 is translocated to the cell surface, it might interact with proteins on an adjacent cell (e.g. integrins or syndecan). In analogy to related proteases, ADAM10 activity at the plasma membrane might be regulated by intracellular interactors that induce phosphorylation or more complex signaling alterations. Importantly, several ADAM proteases (including ADAM10) apparently require positioning into defined membrane platforms (e.g. lipid rafts or tetraspanin platforms) to get into proximity to their substrates. If substrate cleavage occurs, the released soluble ectodomain of the substrate can act in an autocrine or paracrine fashion. Interestingly, some ADAM proteases (e.g. ADAM12) are recycled in a clathrin-dependent manner, supported by SH3 domain adaptors such as Grb-2. Moreover, it was reported that ADAM10 itself is proteolytically processed by ADAM9 and 15 and that the remaining C-terminal fragments (CTFs) are subjected to intramembrane proteolysis by γ-secretase releasing an isolated intracellular domain (ICD) into the cytosol. Importantly, if available for protein-protein interactions, the intracellular region of ADAM10 containing the SH3 binding sites may affect all different aspects from intracellular transport, via plasma membrane positioning and activation to recycling or translocation and degradation of the CTFs or ICDs. The presented model was modified based on a cartoon by Seals and Courtneidge (Genes Dev. 2003, 17:7–30) [ref. [8] of the main manuscript] to highlight potential sites of action of SH3 domain interactors identified in the present study. For more detailed information concerning the putative function of individual binding partners, we refer to the Results and Discussion part of the main manuscript.
(TIF)

Appendix S1 Pull down analyses.
(DOCX)
