# Supplementary figures and images for: Identification of SH3 Domain Proteins Interacting with the Cytoplasmic Tail of the A Disintegrin and Metalloprotease 10 (ADAM10)
Source: PLoS One. 2014 Jul 18;9(7):e102899. doi: 10.1371/journal.pone.0102899 (PMC4103893; doi:10.1371/journal.pone.0102899)

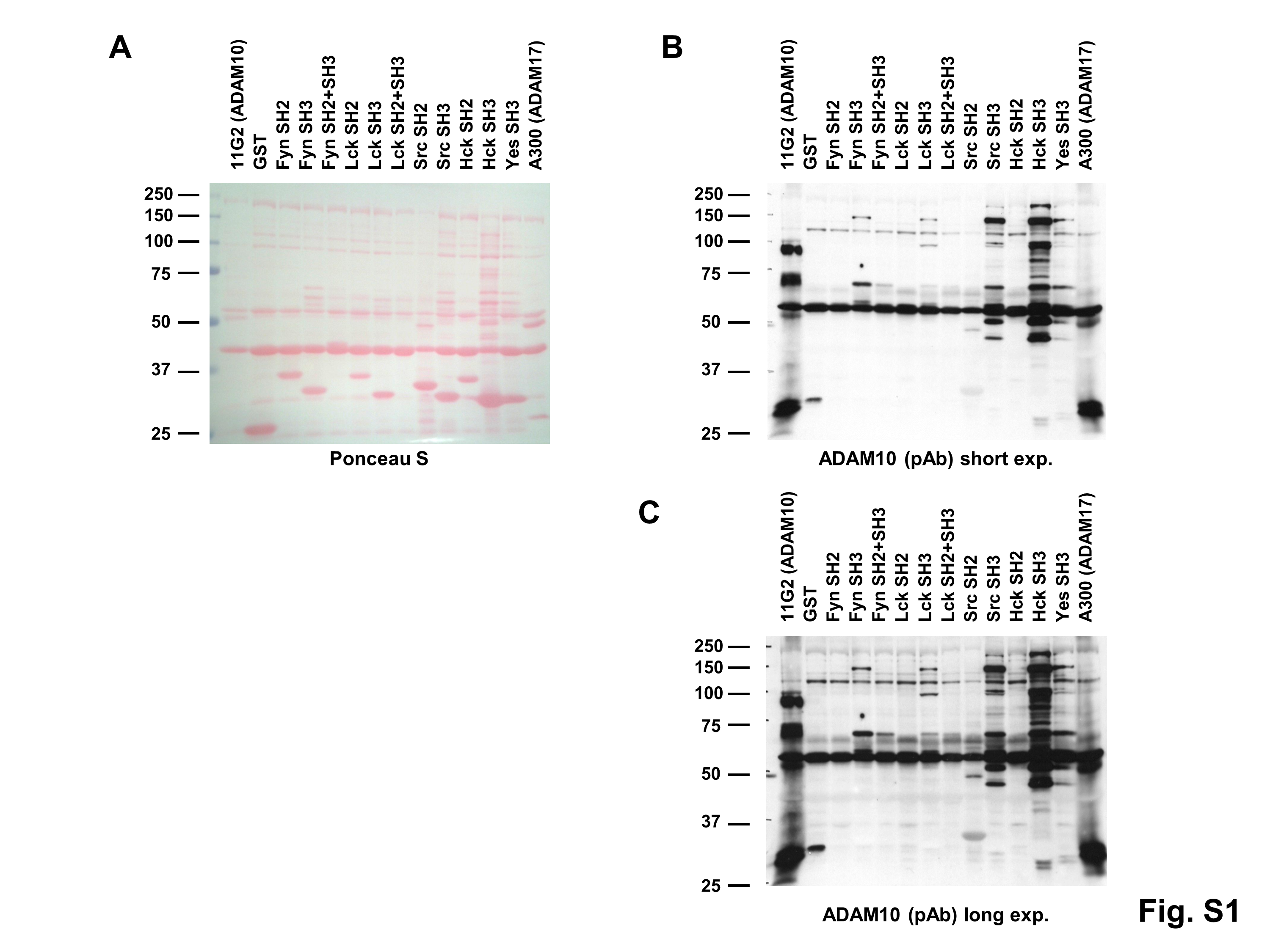

Supplement: Figure S1 — Immunoprecipitation and pull down from human PHA blasts – Src related kinases. PHA-stimulated T cells were lysed in NP40 lysis buffer containing EDTA and protease and phosphatase inhibitors. Immunoprecipitations were performed from 1 ml of cell lysate (equivalent to 50×106 cells) using 2 µg/ml of the indicated anti-ADAM10 or anti-ADAM17 antibodies. Precipitations with GST as a control or GST fusion proteins containing SH2 and/or SH3 domains of the Src-related kinases Fyn, Lck, Src, Hck and Yes were done using 25 µg/ml lysate of the respective fusion proteins. (A) Ponceau S staining following Western transfer. (B) ADAM10 immunoblot using the polyclonal anti-ADAM10 antibody (“animal 1”) - short exposure time. (C) ADAM10 immunoblot using the polyclonal anti-ADAM10 antibody (“animal 1”) - long exposure time. (TIF) [file pone.0102899.s001.tif]

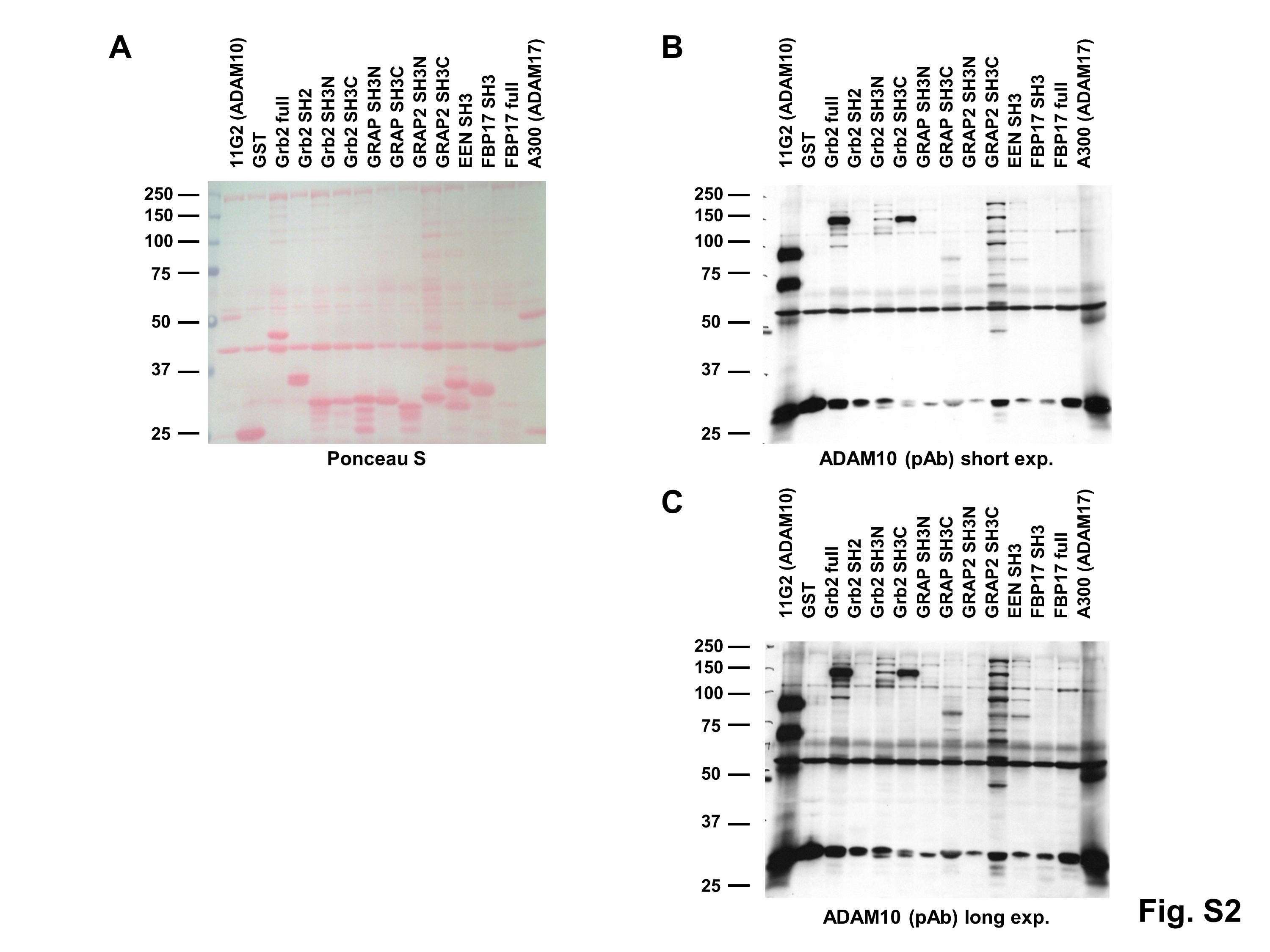

Supplement: Figure S2 — Immunoprecipitation and pull down from human PHA blasts – Grb-2 related adaptor proteins, EEN and FBP17. PHA-stimulated T cells were lysed in NP40 lysis buffer containing EDTA and protease and phosphatase inhibitors. Immunoprecipitations were performed from 1 ml cell lysate (equivalent to 50×106 cells) using 2 µg/ml of the indicated anti-ADAM10 or anti-ADAM17 antibodies. Precipitations with GST as a control or GST fusion proteins containing SH2 and/or SH3 domains or full length proteins of Grb2, GRAP, GRAP2, EEN and FBP17 were done using 25 µg/ml lysate of the respective fusion proteins. (A) Ponceau S staining following Western transfer. (B) ADAM10 immunoblot using the polyclonal anti-ADAM10 antibody (“animal 1”) - short exposure time. (C) ADAM10 immunoblot using the polyclonal anti-ADAM10 antibody (“animal 1”) - long exposure time. (TIF) [file pone.0102899.s002.tif]

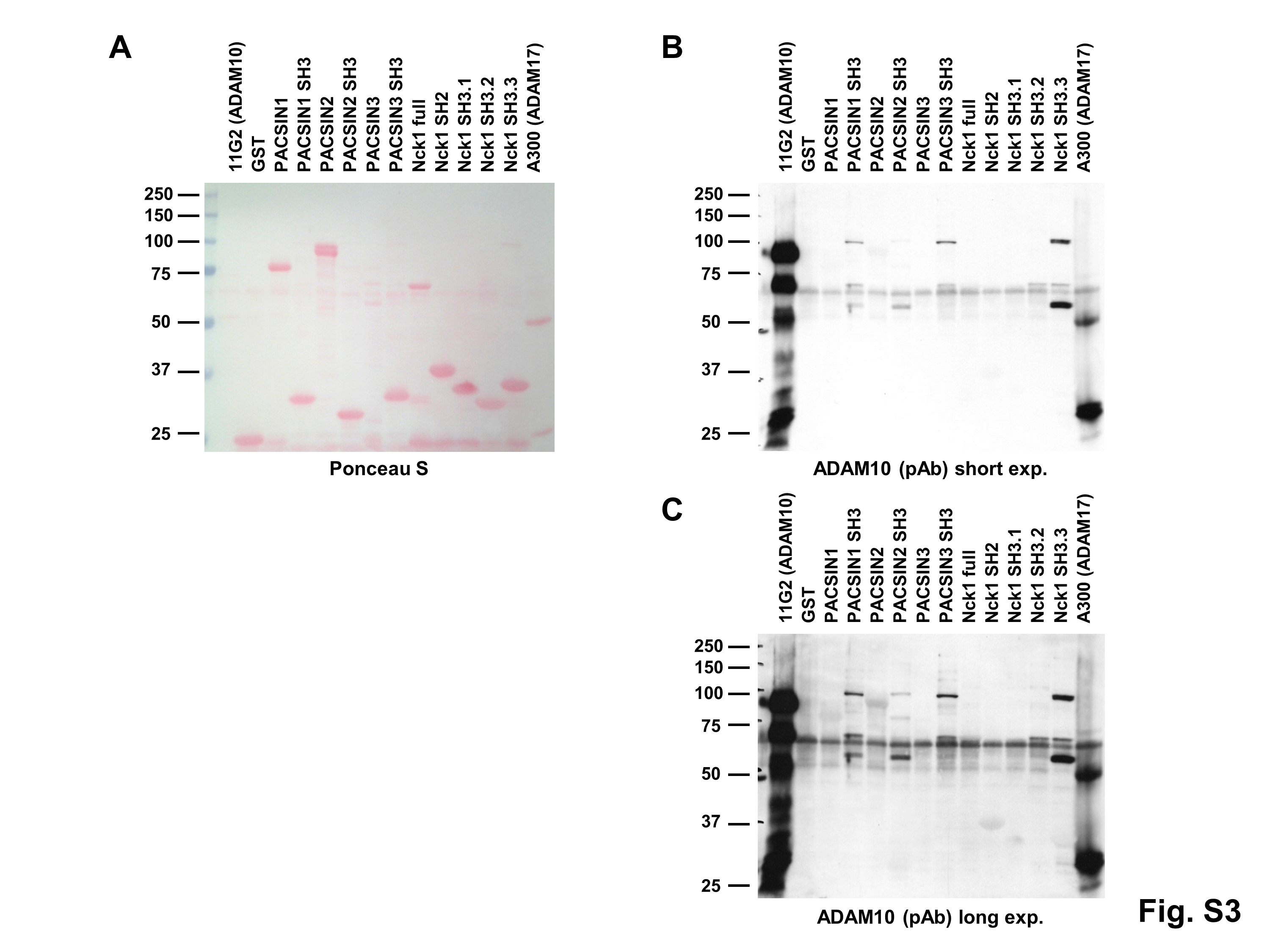

Supplement: Figure S3 — Immunoprecipitation and pull down from human Jurkat cells (JE6-1) – PACSINs and Nck1. Jurkat cells were lysed in NP40 lysis buffer containing EDTA and protease and phosphatase inhibitors. Immunoprecipitations were performed from 1 ml cell lysate (equivalent to 50×106 cells) using 2 µg/ml of the indicated anti-ADAM10 or anti-ADAM17 antibodies. Precipitations with GST as a control or GST fusion proteins containing SH2 and/or SH3 domains or full length proteins of PACSINs or Nck1 were done using 25 µg/ml lysate of the respective fusion proteins. (A) Ponceau S staining following Western transfer. (B) ADAM10 immunoblot using the polyclonal anti-ADAM10 antibody (“animal 1”) - short exposure time. (C) ADAM10 immunoblot using the polyclonal anti-ADAM10 antibody (“animal 1”) - long exposure time. (TIF) [file pone.0102899.s003.tif]

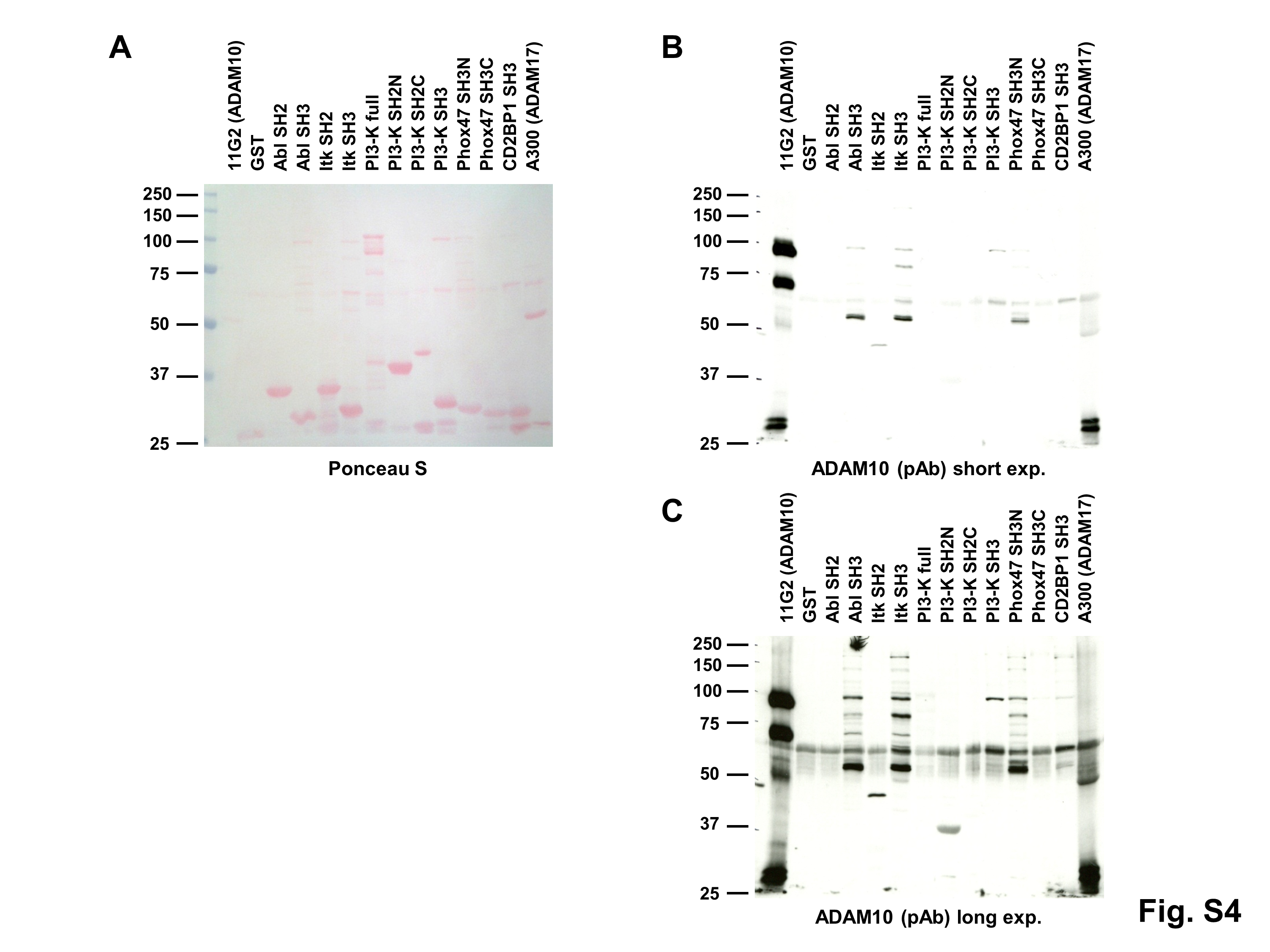

Supplement: Figure S4 — Immunoprecipitation and pull down from human Jurkat cells (JE6-1) – Abl, Itk, PI 3K, phox47 and CD2BP1. Jurkat cells were lysed in NP40 lysis buffer containing EDTA and protease and phosphatase inhibitors. Immunoprecipitations were performed from 1 ml cell lysate (equivalent to 50×106 cells) using 2 µg/ml of the indicated anti-ADAM10 or anti-ADAM17 antibodies. Precipitations with GST as a control or GST fusion proteins containing SH2 and/or SH3 domains or full length proteins of Abl, Itk, PI 3K, phox47 or CD2BP1 were done using 25 µg/ml lysate of the respective fusion proteins. (A) Ponceau S staining following Western transfer. (B) ADAM10 immunoblot using the polyclonal anti-ADAM10 antibody (“animal 1”) - short exposure time. (C) ADAM10 immunoblot using the polyclonal anti-ADAM10 antibody (“animal 1”) - long exposure time. (TIF) [file pone.0102899.s004.tif]

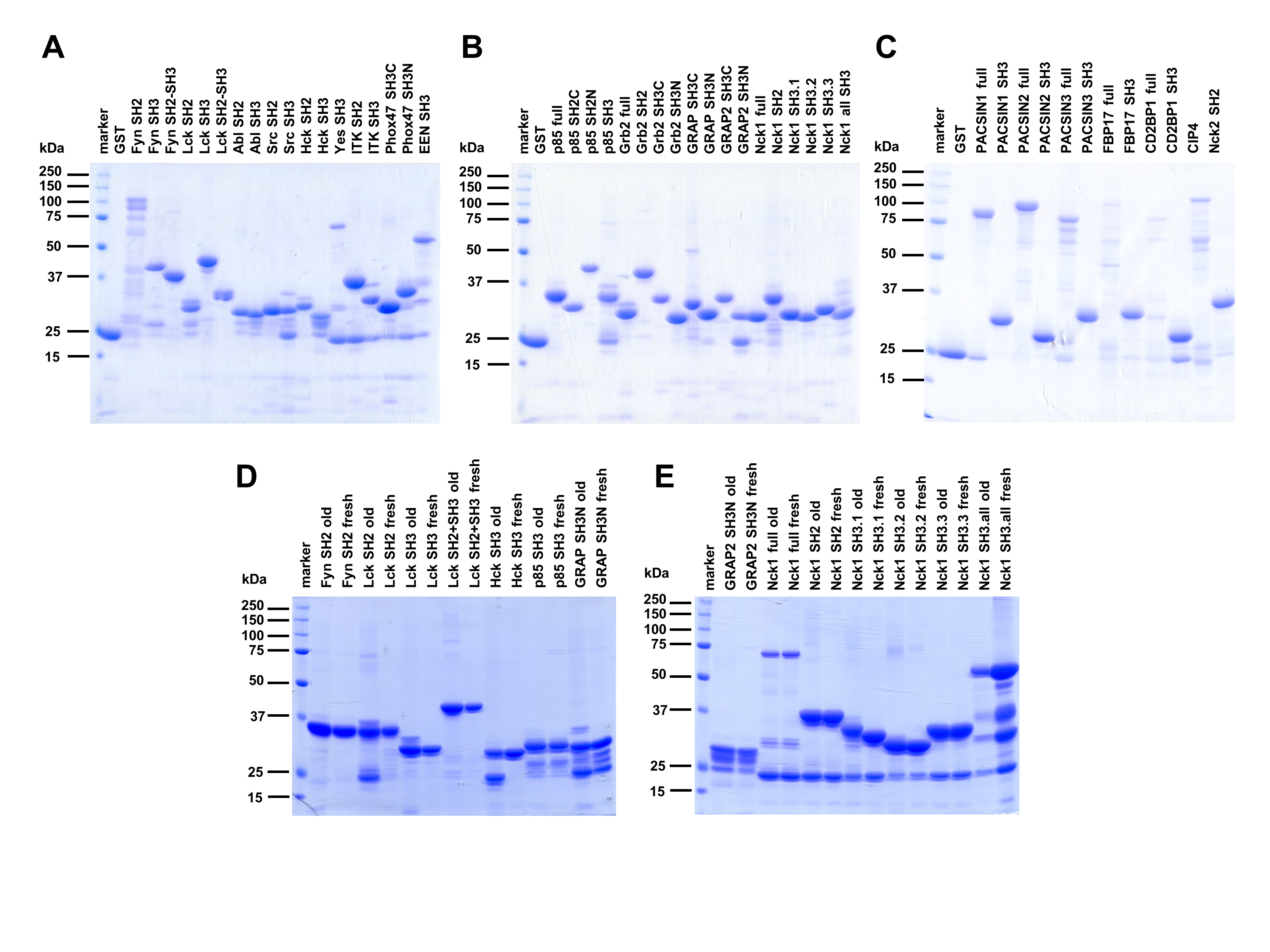

Supplement: Figure S5 — Coomassie blue staining of fusion proteins used for pull down experiments. (A-C) Prior to the precipitations from cell lysates as depicted in the supplementary Figures S1–S4, 25 µg of all fusion proteins were checked after separation by SDS-PAGE for degradation by in-gel-staining with coomassie blue. (D–E) Potentially degraded (“old”) fusion proteins were compared to freshly thawed material (“fresh”) and replaced if necessary. (TIF) [file pone.0102899.s005.tif]

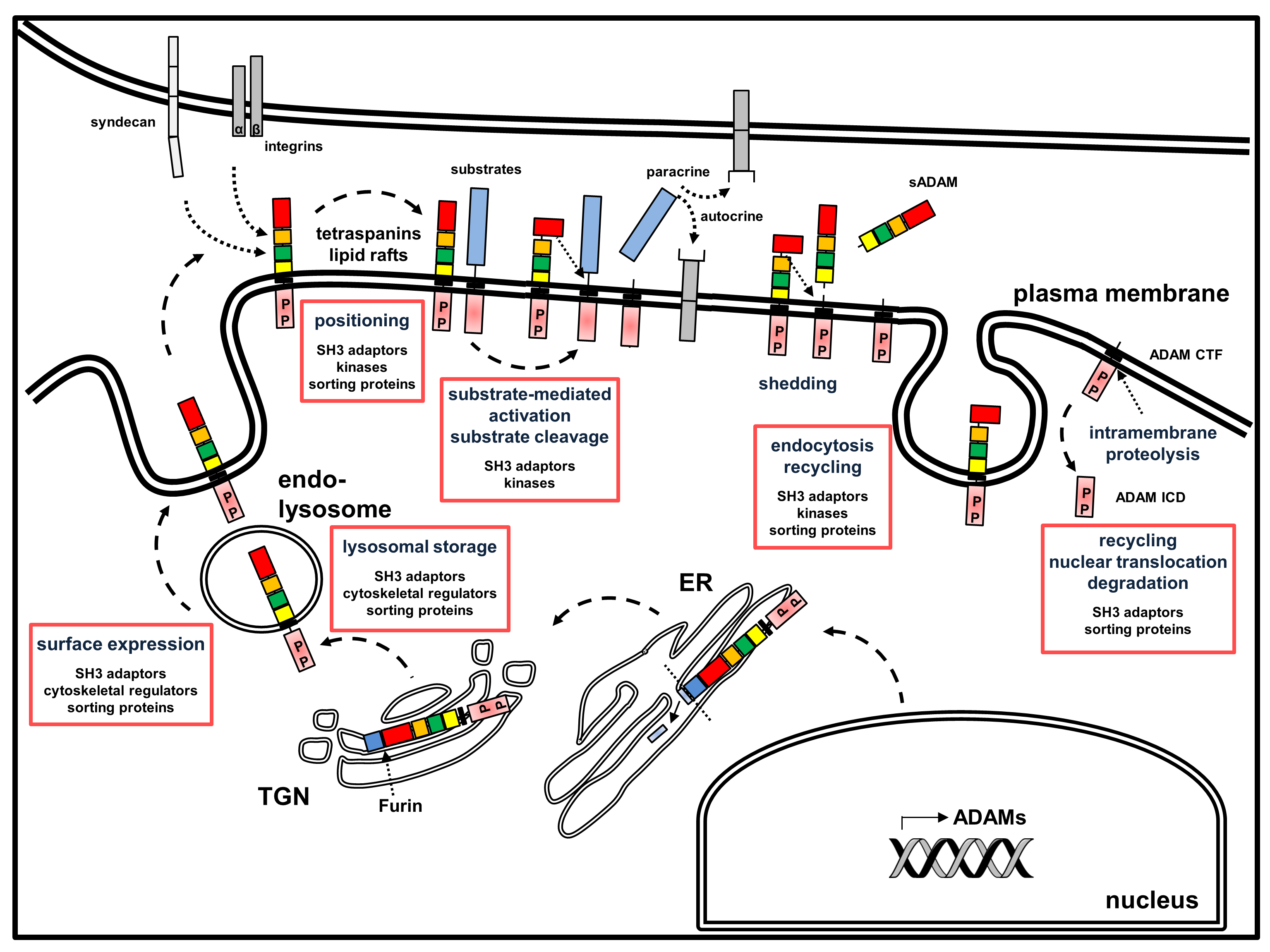

Supplement: Figure S6 — The complex ADAM biology – a hypothetical model depicting putative regulation by SH3 domain proteins. ADAM proteases are synthesized in the rough endoplasmatic reticulum (ER) with a signal peptide that is removed when entering the trans-Golgi network (TGN). It is believed that the pro-domain is cleaved off in the Golgi compartment by protein convertases such as furin. In certain cell types, ADAM10 activity has been associated with a lysosomal compartment. It is, however, not clear whether this organelle association is involved in storage or translocation of the protease or whether the protease exerts intracellular activity in an endo-lysosomal compartment. When ADAM10 is translocated to the cell surface, it might interact with proteins on an adjacent cell (e.g. integrins or syndecan). In analogy to related proteases, ADAM10 activity at the plasma membrane might be regulated by intracellular interactors that induce phosphorylation or more complex signaling alterations. Importantly, several ADAM proteases (including ADAM10) apparently require positioning into defined membrane platforms (e.g. lipid rafts or tetraspanin platforms) to get into proximity to their substrates. If substrate cleavage occurs, the released soluble ectodomain of the substrate can act in an autocrine or paracrine fashion. Interestingly, some ADAM proteases (e.g. ADAM12) are recycled in a clathrin-dependent manner, supported by SH3 domain adaptors such as Grb-2. Moreover, it was reported that ADAM10 itself is proteolytically processed by ADAM9 and 15 and that the remaining C-terminal fragments (CTFs) are subjected to intramembrane proteolysis by γ-secretase releasing an isolated intracellular domain (ICD) into the cytosol. Importantly, if available for protein-protein interactions, the intracellular region of ADAM10 containing the SH3 binding sites may affect all different aspects from intracellular transport, via plasma membrane positioning and activation to recycling or translocation and degrada [file pone.0102899.s006.tif]
