# Supplementary material for: Identification of SH3 Domain Proteins Interacting with the Cytoplasmic Tail of the A Disintegrin and Metalloprotease 10 (ADAM10)
Source: PLoS One. 2014 Jul 18;9(7):e102899. doi: 10.1371/journal.pone.0102899 (PMC4103893; doi:10.1371/journal.pone.0102899)
Supplement: Appendix S1 — Pull down analyses. (DOCX) [file pone.0102899.s007.docx]

**Pull down analyses**

Pull down experiments were performed from PHA blasts (supplementary Figures S1 and S2) or Jurkat cells (JE6-1, supplementary Figures S3 and S4). The cells were washed twice with cold PBS and 50x10^6^ cells were lysed in 1 ml of cold lysis buffer containing 1% NP40, 5 mM EDTA and protease and phosphatase inhibitors and homogenized using a dounce homogenizer. Following incubation on ice for 20 min, cell debris was removed by centrifugation at 14000 rpm for 10 minutes. For pull down experiments, 1 ml of clear supernatant was added to 25 µg of the respective fusion protein and glutathione sepharose beads (GE Healthcare). Immunoprecipitations were performed with protein G beads (GE Healthcare) and 2 µg of the anti-ADAM10 mAb 11G2 or the isotype-matched anti-ADAM17 mAb A300 [1]. After rotation for 2 h at 4 °C, beads were washed three times with NP40 lysis buffer and precipitates were subjected to SDS-PAGE using 10% gels in a Protean^®^ II xi Cell (Bio-Rad). Following protein transfer to nitrocellulose membranes (GE Healthcare), Ponceau S staining was performed and recorded (supplementary Fig. S1-S4, panel A). The blots were then washed in TBS-T and blocked with 5% (v/v) BSA in TBS-T for 1 hour. Membranes were then incubated with polyclonal anti-ADAM10 antiserum (1:1000 in TBS-T) for 1 hour at room temperature, washed three times with TBS-T and subsequently incubated for 45 minutes with the horseradish peroxidase-conjugated donkey anti-rabbit secondary antibody (1:7500 in TBS-T) at room temperature. After three additional washes in TBS-T, blots were developed using ECL reagents and films from GE Healthcare

Reference

1. Trad A, Hedemann N, Shomali M, Pawlak V, Grötzinger J, Lorenzen I (2011) Development of sandwich ELISA for detection and quantification of human and murine a disintegrin and metalloproteinase17. J Immunol Methods 371: 91-96.
